# Analysis of the insertion/deletion polymorphism of the human angiotensin converting enzyme (*ACE*) gene in patients with renal cancer

**DOI:** 10.1054/bjoc.1999.0962

**Published:** 2000-01-18

**Authors:** B A Usmani, M Janeczko, R Shen, M Mazumdar, C N Papandreou, D M Nanus

**Affiliations:** Genitourinary Oncology Research Laboratory and Department of Biostatistics, Memorial Sloan-Kettering Cancer Center, New York, NY, USA; Departments of Medicine and Urology, Weill Medical College of Cornell University, New York, NY, 10021, USA

**Keywords:** ACE, polymorphism, kidney, renal cancer

## Abstract

The angiotensin I-converting enzyme (ACE) contains an insertion/deletion (I/D) polymorphism, with the DD genotype associated with benign renal diseases. The distribution frequencies of the D and I alleles, and the DD, DI and II genotypes were determined in DNA extracted from kidney tissues of 58 renal cancer patients. The observed frequencies in patients who develop renal cancer was not significantly different than the normal population. © 2000 Cancer Research Campaign


British Journal of Cancer (2000) **82**(3), 550–552
© 2000 Cancer Research Campaign
DOI: 10.1054/ bjoc.1999.0962, available online at http://www.idealibrary.com on IDEAL®

### Analysis of the insertion/deletion polymorphism of the human angiotensin converting enzyme (*ACE*) gene in patients with renal cancer

**BA Usmani[1], M Janeczko[1,4], R Shen[1,4], M Mazumdar[2], CN Papandreou[1] and DM Nanus[1,3,4]**

[1]Genitourinary Oncology Research Laboratory and [2]Department of Biostatistics, Memorial Sloan-Kettering Cancer Center, New York, NY, USA; Departments of [3]Medicine and [4]Urology, Weill Medical College of Cornell University, New York, NY 10021, USA

**Summary** The angiotensin I-converting enzyme (ACE) contains an insertion/deletion (I/D) polymorphism, with the DD genotype associated with benign renal diseases. The distribution frequencies of the D and I alleles, and the DD, DI and II genotypes were determined in DNA extracted from kidney tissues of 58 renal cancer patients. The observed frequencies in patients who develop renal cancer was not significantly different than the normal population. © 2000 Cancer Research Campaign

**Keywords:** ACE; polymorphism; kidney; renal cancer

The angiotensin I-converting enzyme (ACE) is found in the plasma membrane of vascular endothelial cells and on a variety of extravascular tissues, while the soluble form of ACE circulates in plasma. The inter-individual variability of human plasma and cellular ACE levels is predominantly under the influence of a genetic polymorphism in the *ACE* gene (Alhenc-Gelas et al, 1997), which corresponds to the presence or absence of a 287-bp Alu-type sequence located in intron 16. Individuals homozygous for the insertion (I genotype) have lower plasma and membrane-bound ACE levels than those homozygotes without the insertion (deletion, or D genotype), while heterozygotes display intermediate levels (DI geneotype) (Rigat et al, 1990). The DD genotype is significantly associated with diabetic nephropathy in type I diabetes (Marre et al, 1994), progressive renal dysfunction leading to chronic renal failure in IgA nephropathy (Yoshida et al, 1995), renal artery stenosis (Missouris et al, 1996) and end-stage renal failure in polycystic kidney disease (Baboolal et al, 1997). We considered whether the DD genotype occurs with an increased frequency in patients developing renal cell carcinoma. We therefore analysed DNA derived from normal kidney tissue of 58 patients who underwent nephrectomy for primary renal tumours for the prevalence of the I and D alleles, and for the incidence of the DD genotype.

### MATERIALS AND METHODS

### Tissue specimens and PCR analysis

Surgically removed renal tumour specimens were obtained from 58 random patients undergoing radical nephrectomy at Memorial Sloan-Kettering Cancer Center (MSKCC) and processed as described (Nanus et al, 1990). Polymerase chain reaction (PCR) was performed on DNA derived from the tumours using gene-specific oligonucleotide primers as previously reported (Albino et al, 1991; Nanus et al, 1993a). A single PCR product of 190 bp product was genotyped DD, of 490 bp product was genotyped II, and if both 190 bp and 490 bp were present the sample was genotyped DI (Rigat et al, 1990). Any DNA sample genotyped DD underwent a second amplification with a nested primer to confirm the genotype (Shanmugan et al, 1993). Amplification of the *ACE* gene used the following amplimers: sense 5′-CTGGAGAC-CACTCCCATCCTTTCT-3′ and anti-sense 5′-GATGTG-GCCATCACATTCGTCAGAT-3′ (Rigat et al, 1992). The DD genotypes were confirmed using a nested sense primer 5′-TTTGA-GACGGAGTCTCGCTC-3′ as described (Shanmugan et al, 1993). A $\chi^2$ goodness of fit test was performed to determine if the observed proportion match with the control proportion (Armitage and Berry, 1987). Two-sided *P*-values are reported.

### RESULTS

DNA was extracted from normal kidney obtained at nephrectomy from 58 patients undergoing surgical resection of primary renal tumours. There were of 36 males and 22 females with a median age of 64 (range 32–83). Histological review revealed 46 clear cell carcinomas and 12 non-clear cell histologies (four papillary carcinomas, four chromophobe carcinomas, four oncocytomas). DNA was amplified using gene-specific primers for intron 16. Representative PCR results are illustrated in Figure 1. The frequency of the ACE D and I alleles for all 58 patients was 0.612 and 0.388 respectively. This frequency was similar to that observed in a control population (Table 1). The observed frequencies of 0.362, 0.50 and 0.138 for the DD, DI and II genotypes respectively were not significantly different from the frequencies predicted by Hardy–Weinberg equilibrium ($\chi^2$ = 2.01; $P$ = 0.4). Subset analysis of the distribution of the D and I alleles in clear-cell versus non-clear cell histology revealed a 75% incidence of the D allele in patients with non-clear cell renal tumours ($\chi^2$ = 3.7, $P$ = 0.06), however, there were only 12 samples in this cohort.

*Received 20 April 1999*
*Revised 12 August 1999*
*Accepted 25 August 1999*

*Correspondence to:* DM Nanus, The New York Presbyterian Hospital–Cornell Medical Center, 520 E. 70th Street, ST-341, New York, NY 10021, USA

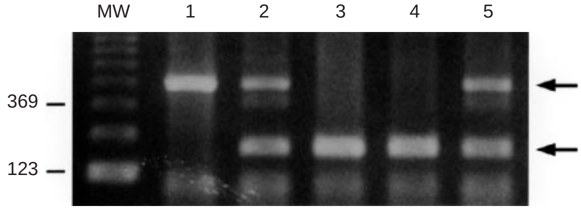

**Figure 1** PCR amplification of ACE alleles. Representative data of DNA extracted from normal kidney tissue amplified using insertion-specific primers for intron 16, and the PCR product separated on an agarose gel. Representative samples shown: I genotype (lane 1); DI genotype (lanes 2 and 5); and DD genotype (lanes 3 and 4). MW = molecular weight marker

**Table 1** Distribution of ACE alleles and genotypes in DNA of patients with kidney cancer

| | CC[a] (*n* = 46) | Non-CC (*n* = 12) | All (*n* = 58) | Controls[c] (*n* = 553) |
|---|---|---|---|---|
| ACE alleles | | | | |
| D | 53 (0.576)[b] | 18 (0.75) | 71 (0.612) | 0.555 |
| I | 39 (0.424) | 6 (0.25) | 45 (0.388) | 0.445 |
| ACE genotypes | | | | |
| DD | 15 (0.341) | 6 (0.5) | 21 (0.362) | 0.284 |
| DI | 23 (0.522) | 6 (0.5) | 29 (0.5) | 0.533 |
| II | 8 (0.182) | 0 | 8 (0.138) | 0.183 |

[a]CC = clear cell carcinoma; Non-CC = non-clear cell. [b]Number (fequency). [c]Other population control as described (Schachter et al, 1994).

### DISCUSSION

The distribution of the ACE D and I alleles and the ACE genotypes in 58 patients diagnosed with renal cell carcinoma was similar to that observed in the normal population, and did not associate with the development of renal cancer. These data contrast multiple studies in which the DD polymorphism, which results in elevated plasma and membrane bound ACE, is associated with the development of benign renal diseases (Costerousse et al, 1997). Elevated ACE levels lead to increased angiotensin II production and bradykinin inactivation, and it is the increase in angiotensin II that is believed to contribute to the numerous pathological states associated with the DD genotype. Recent data indicate that angiotensin II functions at the cellular level as a growth factor (Wolf and Neilson, 1993; Wolf, 1995) and is essential for normal growth and development of renal tubules (Alcorn and Maric, 1996).

Despite that the current study which did not identify an increased frequency of the DD polymorphism does not implicate elevated ACE and consequently increased angiotensin II levels in the development of renal tumours, a number of recent studies do suggest that the renin-angiotensin system plays a role in renal carcinogenesis. Angiotensin II can induce the synthesis of basic fibroblast growth factor (bFGF) (Itoh et al, 1993) which has been implicated in the development and progression of renal cancers (Nanus et al, 1993b). The ACE inhibitor Captopril significantly reduces the tumour size of renal cancer xenografts in athymic mice in a dose-dependent fashion (Hii et al, 1998). We have shown that overexpression of aminopeptidase A, which converts angiotensin II to angiotensin III, in renal cancer cells inhibits anchorage-independent growth and tumorigenecity (unpublished data). Finally, one recent study suggests that long-term use of ACE inhibitors may protect against cancer (Lever et al, 1998). Taken together, these data begin to implicate ACE and its substrates including angiotensin II in the pathogenesis of renal cancers, and indicate that the involvement of the tissue renin-angiotensin system in this disease warrants further study.

In summary, the distribution of the ACE polymorphism in 58 patients with renal cell carcinomas is not significantly different than the expected incidence in patients without renal cancers. These data suggest that elevated ACE levels do not predispose to the development of renal tumours. Further studies are needed to define the involvement of the renin-angiotensin system in renal carcinogenesis.

### ACKNOWLEDGEMENTS

The authors thank Ms Jennifer Wilkes for expert technical assistance, Dr Victor Reuter for review of pathology specimens and Dr Pierre Corvol for useful discussions. This study was supported in part by NIH Grant CA 57475.

### REFERENCES

Albino AP, Davis BM and Nanus DM (1991) Induction of growth factor RNA expression in human malignant melanoma: markers of transformation. *Cancer Res* **51:** 4815–4820

Alcorn DMJ and Maric C (1996) Angiotensin receptors and development – the kidney. *Clin Exp Pharmacol Physiol* **3:** S88–92

Alhenc-Gelas F, Costerousse O and Danilov S (1997) Genetic and physiological aspects of angiotensin I-converting enzyme. In: *Cell-surface Peptidases in Health and Disease*, Kenny AJ and Boustead CM (eds), pp. 119–135. Bios Scientific: Oxford

Armitage P and Berry G (1987) *Statistical Methods in Medical Research*, pp. 405–407. Blackwell Scientific: Oxford

Baboolal K, Ravine D, Daniels J, Williams N, Holmans P, Coles GA and Williams JD (1997) Association of the angiotensin I converting enzyme gene deletion polymorphism with early onset of ESRF in PKD1 adult polycystic kidney disease. *Kidney Int* **52:** 607–613

Costerousse O, Danilov S and Alhenc-Gelas F (1997) Genetics of angiotensin I-converting enzyme. *Clin Exp Hyperten* **19**: 659–669

Hii SI, Nicol DL, Gotley DC, Thompson LC, Green MK and Jonsson JR (1998) Captopril inhibits tumour growth in a xenograft model of human renal cell carcinoma. *Br J Cancer* **77**: 880–883

Itoh H, Mukoyama M, Pratt RE, Gibbons GH and Dzau VJ (1993) Multiple autocrine growth factors modulate vascular smooth muscle cell growth response to angiotensin II. *J Clin Invest* **91**: 2268–2274

Lever AF, Hole DJ, Gillis CR, McCallum IR, McInnes GT, MacKinnon PL, Meredith PA, Murray LS, Reid JL and Robertson JWK (1998) Do inhibitors of angiotensin-I-converting enzyme protect against the risk of cancer? *Lancet* **352**: 179–184

Marre M, Bernadet P, Gallois Y, Savagner F, Guyene TT, Hallab M, Cambien F, Passa P and Alhenc-Gelas F (1994) Relationships between angiotensin I converting enzyme gene polymorphism, plasma levels, and diabetic retinal and renal complications. *Diabetes* **43**: 384–388

Missouris CG, Barley J, Jeffery S, Carter ND, Singer DR and MacGregor GA (1996) Genetic risk for renal artery stenosis: association with deletion polymorphism in angiotensin 1-converting enzyme gene. *Kidney Int* **49**: 534–537

Nanus DM, Mentle IR, Motzer RJ, Bander NH and Albino AP (1990) Infrequent ras oncogene point mutations in renal cell carcinoma. *J Urol* **143**: 175–178

Nanus DM, Engelstein D, Gastl GA, Gluck L, Vidal MJ, Morrison M, Finstad CL, Bander NH and Albino AP (1993*a*) Molecular cloning of the human kidney differentiation antigen gp160: human aminopeptidase A. *Proc Natl Acad Sci USA* **90**: 7069–7073

Nanus DM, Schmitz-Drager BJ, Motzer RJ, Lee AC, Vlamis V, Cordon-Cardo C, Albino AP and Reuter VE (1993*b*) Expression of basic fibroblast growth factor in primary human renal tumors: correlation with poor survival. *J Natl Cancer Inst* **85**: 1597–1599

*© 2000 Cancer Research Campaign*

Rigat B, Hubert C, Alhenc-Gelas F, Cambien F, Corvol P and Soubrier F (1990) An insertion/deletion polymorphism in the angiotensin I-converting enzyme gene accounting for half the variance of serume enzyme levels. *J Clin Invest* **86**: 1343–1346
Rigat B, Hubert C, Corvo P and Soubrier F (1992) PCR detection of the insertion/deletion polymorphism of the. *Nucleic Acids Res* **20**: 1433–1433
Schachter F, Faure-Delanef L, Guenot F, Rouger H, Froguet P, Lesueur-Ginot L and Cohen D (1994) Genetic associations with human longevity at the APOE and ACE loci. *Nat Genet* **6**: 29–32
Shanmugan V, Sell KW and Saha BK (1993) Mistyping ACE heterozygotes. *PCR Meth App* **3**: 120–121
Wolf G (1995) Angiotensin as a renal growth promoting factor. *Adv Exp Med Biol* **377**: 225–236
Wolf G and Neilson EG (1993) Angiotensin II as a renal growth factor. *J Am Soc Nephrol* **3**: 1531–1540
Yoshida H, Mitarai T, Kawamura T, Kitajima T, Miyazaki, Nagasawa R, Kawaguchi Y, Kubo H, Ichikawa I and Sakai (1995) Role of the deletion of polymorphism of the angiotensin converting enzyme gene in the progression and therapeutic responsiveness of IgA nephropathy. *J Clin Invest* **96**: 2162–2169

*© 2000 Cancer Research Campaign*
